# Laparoscopically Assisted Percutaneous Inguinal Ring Closure for Resolution of Inguinal/Scrotal Hernias in Rams: Cadaveric Study and Three Cases Report

**DOI:** 10.3390/ani13050836

**Published:** 2023-02-24

**Authors:** Francisco J. Vázquez, Antonio Romero, Sara Fuente, Laura Barrachina, Arantza Vitoria

**Affiliations:** 1 Hospital Veterinario (HVUZ), Universidad de Zaragoza, 50013 Zaragoza, Spain; 2 Departamento de Patología Animal, Facultad de Veterinaria, Universidad de Zaragoza, 50013 Zaragoza, Spain; 3 Instituto Agroalimentario de Aragón (IA2), 50013 Zaragoza, Spain; 4 Departamento de Anatomía, Embriología y Genética Animal, Universidad de Zaragoza, 50013 Zaragoza, Spain

**Keywords:** ram, hernioplasty, inguinal hernia, laparoscopy, percutaneous suture, cadaver, case report

## Abstract

**Simple Summary:**

Indirect inguinal hernia in sheep can reduce the fertility and affected animals may present serious problems that can be life-threatening. The aim of this work is to describe a laparoscopically assisted technique to suture the internal inguinal ring in rams, preserving testicles. In six ram cadavers, both vaginal rings were partially closed. Two methods were tested on each side. The number of sutures required to achieve a satisfactory closure was recorded. After the cadaveric study, the procedure was performed on three rams with inguinal hernia and the occurrence of re-herniation was followed up. In cadavers, the technique could be easily and satisfactorily performed with either of the two systems. In two clinical cases, the procedure was successfully performed without hernia recurrence or alterations in reproductive behavior in the following months. In the third case, problems during laparoscopy prevented hernioplasty and the animal herniated again. This technique can be used as a simple and feasible surgical treatment for rams with inguinal hernias when preservation of the testicles is preferred. Although a larger number of cases are needed to evaluate long-term safety and effectiveness, this technique should be considered as an alternative to castration in rams with inguinal hernia.

**Abstract:**

The aim of this study is to evaluate a laparoscopically assisted percutaneous suture (LAPS) procedure to treat inguinal hernia (IH) while preserving testicles in rams. An ex vivo experiment with six ram cadavers and a report of three clinical cases are discussed. In cadavers, both internal inguinal rings (IIR) were partially closed by LAPS. Two LAPS methods were tested: (1) using a laparoscopic portal closure device and (2) using a suture loop inserted through needles in each IIR. After each procedure, the closure was laparoscopically evaluated and the number of U- sutures was recorded. The procedure was also performed on three client-owned rams with unilateral non-strangulated IH and the occurrence of re-herniation was followed up. In cadavers, LAPS of the IIRs could be easily and satisfactorily performed with either of the two systems, requiring one to three U-sutures per IIR. No differences were observed between the two surgical procedures. In two clinical cases, the procedure was successfully performed without reoccurrence of herniation or alterations in reproductive behavior in the following 3 and 6 months. In the third case, the hernia was reduced but a retroperitoneal emphysema during laparoscopy prevented hernioplasty and the animal herniated again. In conclusion, LAPS of IIR can be used as a simple and feasible treatment to preserve testicles in rams with IH.

## 1. Introduction

Unlike direct inguinal hernia, in which the bowel penetrates directly into the abdominal wall, indirect inguinal hernia (IIH) is characterized by the intestinal bundle protruding through the inguinal canal. This type of hernia accounts for up to 7% of congenital disorders [1] and up to 15% of all hernias in sheep [2]. It is caused by the large size of the internal inguinal ring (IIR) in this species [2]. Despite these hernias usually being non-strangulated, this disorder can reduce the fertility by markedly interfering with the thermoregulatory function of the scrotum and testicles. Furthermore, depending on the herniated intestinal tract, affected animals may present serious digestive problems that can be life-threatening.

Surgical treatment is required when inguinal hernias occur. In humans [3] and horses, laparoscopic techniques have been developed to access the IIR and perform partial hernioplasty, avoiding recurrence of the hernia while preserving the testicle without altering its vascularization, thus allowing the animal to maintain reproductive capacity and, therefore, value [4]. However, this procedure is not usually performed in rams, where the most common approach is herniorrhaphy with orchiectomy [2].

In children, partial closure of the IIR has been described using laparoscopically assisted percutaneous suture (LAPS) techniques. This procedure allows the resolution of the hernia defect in a quick, simple manner and with low recurrence rates, avoiding the use of specific high-cost consumable materials, and, fundamentally, it can be performed without the use of complex laparoscopic techniques [5,6,7]. Recently, a Polish multidisciplinary group of human medicine pediatricians and veterinary surgeons have also reported the use of this technique in the treatment of inguinal hernia in dogs [8] and pigs [9].

Sheep could also benefit from hernioplasty techniques. Although laparoscopy is not so extended in ovine species as in other species, mainly because of the high costs associated, its availability is spreading and is used for some artificial insemination techniques [10] and intrabdominal surgeries [11,12]. Therefore, developing or adapting laparoscopic techniques in sheep that are simple to perform is important to advance the standard point of care in these animals.

The aim of this study was to evaluate the feasibility and efficacy of using a LAPS procedure to treat inguinal/scrotal hernias while preserving testicles in rams. We hypothesized that a modified procedure based on the LAPS technique used in children could be used to achieve partial closure of the vaginal ring in rams with IH. We first conducted a cadaveric study to assess the new technique and to overcome the initial learning curve, and subsequently evaluated the clinical performance of the procedure in three client-owed rams with naturally occurring unilateral non-strangulated IH.

## 2. Materials and Methods

### 2.1. Cadaveric Study

#### 2.1.1. Animals

Six cadavers of adult rams of Rasa Aragonesa breed without signs or history of IH or testicular abnormalities were used, making a total of n = 12 IIRs to test the LAPS technique. The number of animals was based on previous studies describing the development of laparoscopic techniques [13,14].

The rams were euthanized for reasons unrelated to this study. All procedures were carried out under Project Licence PI63/22 approved by the Ethic Committee for Animal Experiments from the University of Zaragoza. The care and use of animals were performed accordingly with the Spanish Policy for Animal Protection RD53/2013, which meets the European Union Directive 2010/63 on the protection of animals used for experimental and other scientific purposes.

#### 2.1.2. LAPS Surgical Procedure

**Figure 1:**
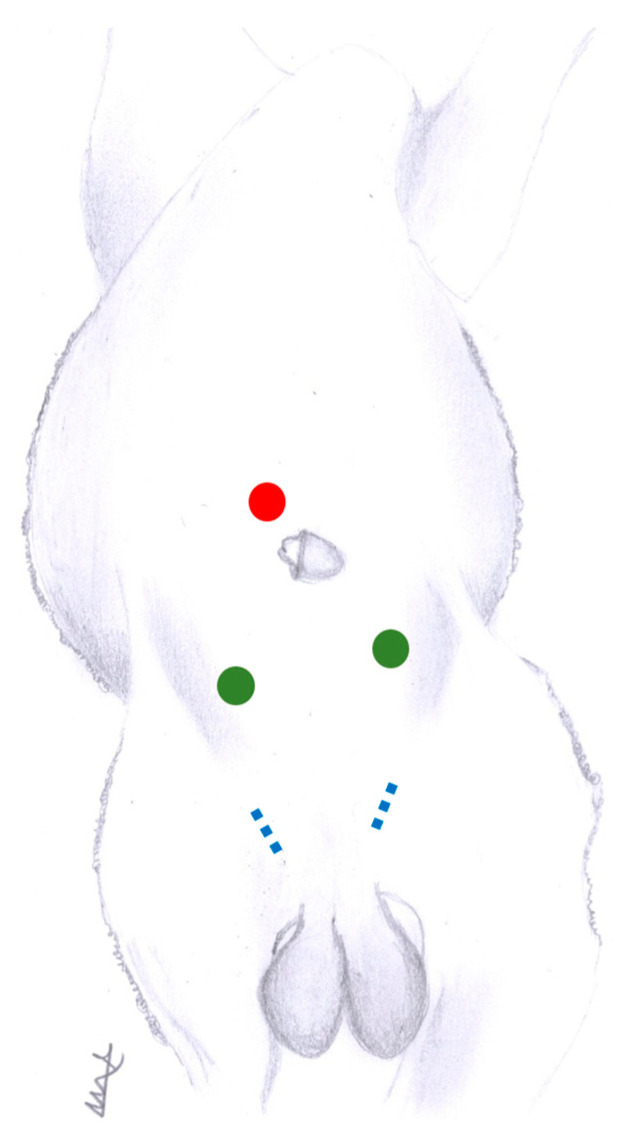
Scheme of laparoscopic portal placement. Red circle: laparoscope portal, placed 5 cm cranial to the prepuce and 5 cm to the right of the midline. Green circles: instrumental portals, placed 10–15 cm cranial to each inguinal ring. Blue dotted lines: inguinal rings.

A wide caudal abdominal area and scrotum were clipped and washed. Cadavers were positioned in dorsal decubitus on a hydraulic surgical table that allowed for Trendelenburg positioning and were positioned as previously described [15]. To create the first laparoscopic portal, a 1.5 cm skin incision was made about 5 cm cranial to the prepuce and 5 cm to the right of the midline. The abdominal wall was elevated with two Backhaus towel clamps while a reusable stainless steel 11 mm diameter and 15 cm long helical optic cannula was inserted. The trocarless cannula was introduced into the abdomen with a rigid 57 or 33 cm 0° laparoscope inside to provide laparoscopic access control. Capnoperitoneum was created with an electronic insufflator until reaching an intra-abdominal pressure of 12–15 mm Hg. Two additional reused plastic disposable trocars and cannulas of 5 or 11 mm diameter were inserted under laparoscopic visualization. These portals were placed 10–15 cranial to each inguinal ring. Regarding needle insertion for placing sutures, an incision of 3–4 cm long was mSW in the skin directly over the inguinal ring. In this way, the knots of the percutaneous sutures remain in the subcutaneous space after the final suturing of the incision (Figure 1).

In all cases, the intervention started on the right side. Two different suture methods were used to perform LAPS on each IIR of each cadaver. The method used on each side was randomly assigned (coin-flipping method performed by the first author). With both methods, sutures should be placed partially closing the flap of the IIR against the abdominal wall, close enough to the cord to prevent the possibility of herniation but not too close as to compress the vas deferens and testicular vessels. Laparoscopic forceps were used to push caudally the testicular cord.

**Figure 2:**
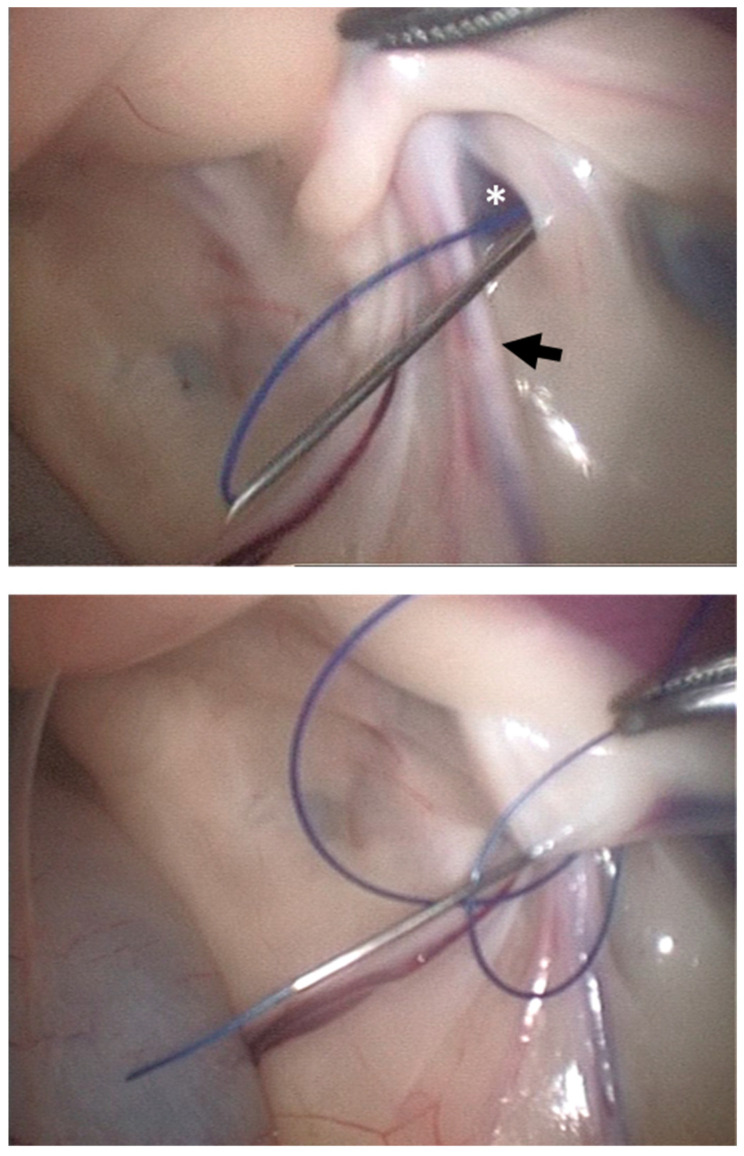
Needle suture loop technique (NSLT) for laparoscopically assisted percutaneous suture of the right internal inguinal ring in a cadaver. (**Top**): 12 G 80 mm needle, with a suture loop through it, inserted through the skin incision into the abdominal cavity under laparoscopic control; asterisk: internal inguinal ring; black arrow: spermatic cord. (**Bottom**): distal end of a 0 USP polyamide suture introduced into the abdomen through the needle and passed into the initial loop with the assistance of laparoscopic forceps.

In the needle suture loop technique (NSLT) method, a 12 G 80 mm needle was used to pass a suture loop and a suture end to perform a U-suture. First, a suture thread (polyamide 0 USP) was passed through the needle and bent back to create the loop. Then, the needle with the suture loop was inserted through the skin incision and introduced in the abdominal cavity under laparoscopic control. Once the needle with the loop was properly placed, the loop was pushed further into the abdominal cavity (Figure 2, top) and retained there with laparoscopic forceps, while the needle was removed. Subsequently, a second needle was inserted in the same way but used to introduce the distal end of a 0 USP polyamide suture through it. From inside the abdomen and using laparoscopic forceps, this suture end was passed through the initial loop (Figure 1, bottom). The needle was then removed and the loop was pulled from outside to create a U-suture stitch around the IIR. A video showing the main steps of the procedure is provided as supplementary material available for this article online (Video S1).

In the second method, the EndoClose Trocar Site Closure Device (Medtronic, Madrid, Spain) was used. This device is designed for the closure of laparoscopic portals in human surgery. It is similar to a Veress needle. The main difference is that the retractable stylet has a notched tip which is used to capture and hold sutures. Similar to the first method, the device with the suture loop was punctured through the skin incision under laparoscopic guidance and laparoscopic forceps were used to retain the suture loop inside the abdomen while the device was withdrawn. With a second puncture of the EndoClose, the initial loop was grasped and externalized to create a U-suture in the IIR.

#### 2.1.3. Assessment of IIR Partial Closure

Proper partial closure of the IIR was confirmed by inserting a blunt laparoscopic trocar through an incision in the scrotum and tunica vaginalis. Prior to tightening the suture, the trocar should easily enter the abdomen (pre-closure control). After knotting the suture on the outside, it was checked whether it was possible to insert the trocar and the outcome was recorded (Supplementary Materials, Video S2). If necessary, an additional suture loop was performed using the same technique as for the first stitch. The number of suture stitches required was recorded.

### 2.2. Clinical Cases

This part of the study was also approved by the Ethic Committee for Animal Experiments from the University of Zaragoza (Project Licence PI66/22) and informed owner (or an authorized agent for the owner) consent was obtained before enrolling any ram.

Three cases of herniated rams referred to our surgical service and subjected to IIR hernioplasty using the LAPS approach were reported. Since owners preferred to preserve the affected testicle, this surgical option was offered. Informed owner consent was obtained after specifically communicating that this disorder could have a hereditary component in case the animals had an intended reproductive use. Follow-up was performed with owners or referrals via telephone calls between 3 to 6 months after surgeries.

## 3. Results

### 3.1. Cadaveric Study

All six carcasses used were of the Rasa Aragonesa breed. The approximate age and weight of cadavers are shown in the Table 1.

The surgical position allowed good visualization of the surgical area, although, in some cases, the cadaver slightly tilted with wedge-folded towels to work on each side. In the initial laparoscopic examination, no hernia or alterations in the IIR were detected in any of the cadavers.

All the procedures were completed successfully. The mean number of U-sutures per IIR was 1.4 (range: 1–3, SD: 0.7). The mean was similar for both methods: 1.5 when using EndoClose (range: 1–3, SD: 0.8) and 1.3 when using NSLT (range: 1–2, SD: 0.5). It should be noted that for the first cadaver, a total of five sutures were necessary, three with EndoClose and two with NSLT. The characteristics of the cadavers and the findings during the procedures are summarized in the Table 1.

Regarding potential adverse events of the surgical technique, complications related to the laparoscopic access, but not to the suture technique itself, were recorded in two cadavers. In one cadaver, the trocar reached the omental bursa during the placement of one of the additional portals. In another cadaver, the rumen was perforated when the first cannula was inserted. To prevent this complication, in two cadavers, part of the gas in the rumen was evacuated before the procedure using a 11 mm, 20 cm locking trocar percutaneously inserted into the rumen.

### 3.2. Clinical Cases

#### 3.2.1. Case 1

**Figure 3:**
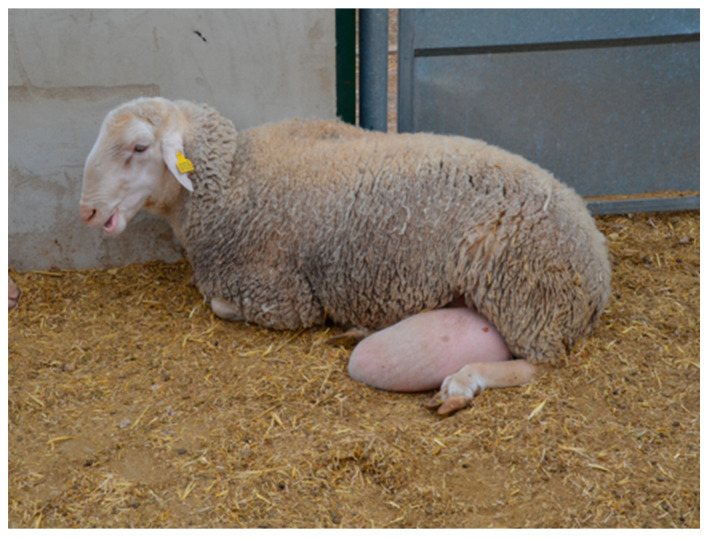
Case 1: Rasa Aragonesa ram with large left scrotal hernia.

A 6/7-year-old (87.5 Kg) Rasa Aragonesa ram was referred with a unilateral left IH. The hernia had appeared some weeks earlier. The ram was intended for breeding. Despite moderate posthitis, the ram had been otherwise healthy and had no history of trauma. General clinical examination and basic parameters were within normal limits. There was visible asymmetry to the scrotum, with left-sided large soft swelling in the scrotum (Figure 3), which could be reduced by palpation in dorsal recumbency. Ultrasonography confirmed the presence of an IH, with intestinal segments in the scrotal sac and hydrocele.

Prior to surgery, food was withheld for 24 h and water was withheld for 12 h. A jugular venous catheter was placed and was used to administer flunixin meglumine (1.1 mg/kg IV, twice daily) and procaine penicillin G (15 mg/kg, once daily, IM). After the induction of general anesthesia, the ram was intubated and connected to an anesthetic machine with a system of positive pressure ventilation and oxygen supply. The ram was placed in dorsal decubitus and secured to the surgical table in the Trendelenburg position. A wide caudal abdominal area and scrotum were clipped and surgically prepared. The testicles were also surgically prepared but kept outside of the surgically draped field, placing the drapes to allow a gloved assistant to manipulate the testicles outside of the sterile area during the surgery, in case external maneuvers were necessary to reduce the hernia.

**Figure 4:**
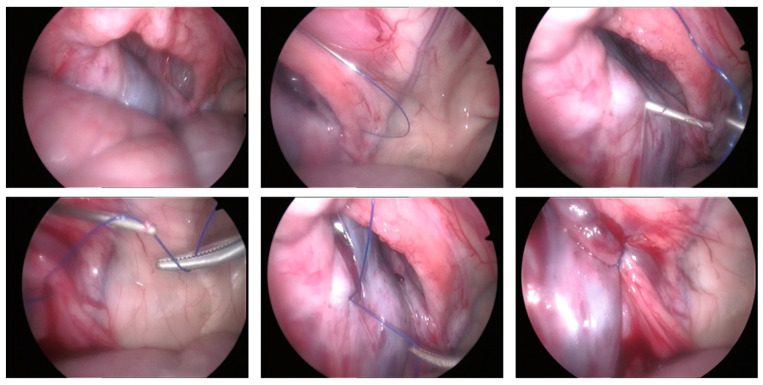
Case 1: Laparoscopically assisted percutaneous suture using the EndoClose device in the first clinical case. (**Top left**): aspect of the left vaginal ring after hernia reduction. (**Top in the middle**): a needle was inserted percutaneously into the ventral aspect of the vaginal ring to introduce a 0-USP polyamide suture through it. (**Top right**): the EndoClose device inserted into the dorsal side of the vaginal ring was used to grasp and exteriorize the suture, completing the U-suture. (**Bottom left**): with the help of laparoscopic forceps, the suture is positioned to be held by the notched tip of the Endoclose. (**Bottom in the middle**): externalizing the suture to create the second U-suture; a slight peritoneal tear can be seen due to the manipulations on the cranial side of the vaginal ring. (**Bottom right**): final laparoscopic appearance of the inguinal ring after placement of three laparoscopically assisted U-sutures.

Access to the abdominal cavity and placement of the laparoscopic portals were performed as described above for the cadaveric study. Laparoscopic examination confirmed the presence of an indirect IH in the left inguinal canal, with a very enlarged IIR. The hernia could be reduced with the combination of external manipulation, laparoscopic atraumatic grasping traction (Endo Clinch II and/or Endo Lung, Covidien), Trendelenburg position and capnoperitoneum (Supplementary Materials, Video S3). The LAPS of the left (herniated) IIR was carried out similarly to that described in the cadaveric study by combining both NSLT and the EndoClose device (Figure 4). Due to the large size of the IIR, three U-suture stitches were necessary for adequate partial closure of IIR. After knotting the percutaneous suture loops in the subcutaneous space, the skin was sutured with monofilar 2/0 USP glycinate in a simple interrupted pattern. The contralateral inguinal ring showed no signs of herniation, but a single percutaneous U-suture was performed using the same method as prophylaxis (Supplementary Materials, Video S4). There were no surgical complications other than a slight tear of the peritoneum adjacent to the IIR that occurred during the manipulations, which did not receive further intervention. The ram recovered from anesthesia without complication. The durations of anesthesia and surgery were 115 and 90 min, respectively.

In the first few days after surgery, there was a marked enlargement of the scrotal sac on the left side, which progressively disappeared in the following weeks. Ultrasonography revealed a moderate amount of hypoechoic fluid but without evidence of a herniated bowel. The incisions healed properly and there was no recurrence of the scrotal hernia. After a 6 months follow-up, there was no recurrence of the hernia and the animal maintained normal sexual behavior.

#### 3.2.2. Case 2

**Figure 5:**
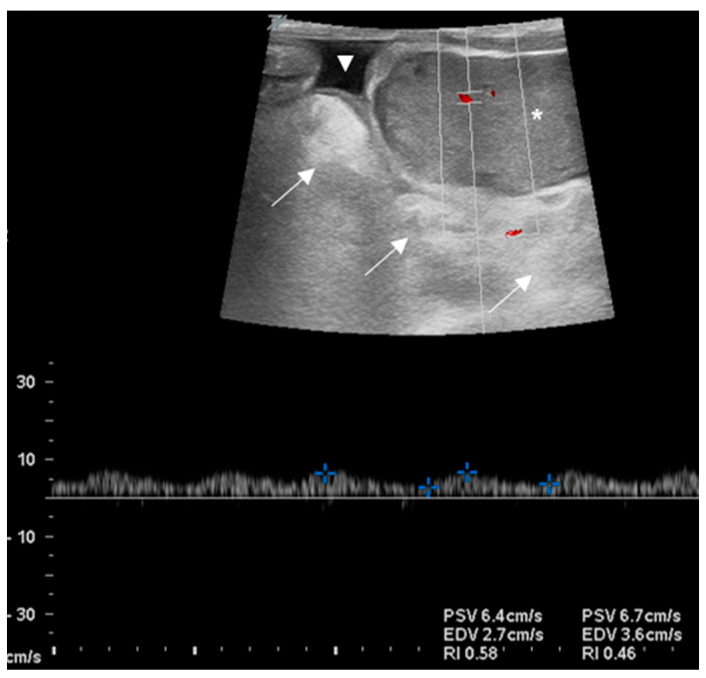
Pulsed Doppler ultrasonography of right testicle in Case 2, showing perfusion parameters measured in an intratesticular artery. Within the ultrasonographic window, the testicle (asterisk) with herniated omentum and the bowel (arrows) and fluid (arrowhead) can be seen.

An 11-month-old (70 Kg) Dorper ram intended for show and breeding was referred with unilateral right IH occurring several weeks earlier, with no relevant changes after initial veterinary assessment. General clinical examination and vital parameters were within normal limits except for an asymmetry with right-sided large soft swelling in the scrotum. The hernia could be reduced externally. Conventional and color Doppler ultrasonography confirmed the presence of an IH, with intestinal segments and omentum in the scrotal sac, hydrocele and with adequate testicular perfusion (Figure 5).

The animal underwent surgery under similar operative and perioperative conditions to Case 1. To reduce the hernia, it was necessary to perform a vigorous external scrotal massage (Supplementary Materials, Video S5). The vaginal ring was remarkably enlarged. During the procedure, after some unsuccessful LAPS attempts in the herniated side, subcutaneous and retroperitoneal emphysema developed, causing visibility to be progressively reduced, and the surgery had to be finished without being able to complete any U-sutures. Two days after reducing the hernia, the scrotal size had reduced considerably compared to admission and ultrasonography revealed a moderate amount of hypoechoic fluid but without evidence of a herniated bowel. The animal returned to his farm and re-intervention was scheduled in 15 days. However, a few days after discharge, the ram re-herniated again and the owner refused further intervention. The ram was sent to the slaughterhouse without the possibility of a necropsy.

#### 3.2.3. Case 3

A 2/3-year-old (75 Kg) Rasa Aragonesa ram intended for breeding was referred for surgical treatment of a mild intermittent unilateral externally reducible left IH that appeared two weeks earlier. The ram was otherwise healthy and had no history of trauma.

The animal was intervened under similar surgical and perioperative conditions to previous cases. The hernia was easily reduced by traction of the intestine from inside the abdomen using laparoscopic forceps. The amount of herniated bowel was more limited than in the two previous cases (Supplementary Materials, Video S6). On this occasion, a first U-suture was performed in the herniated IIR using a polyamide 1 USP suture with its own traumatic 30 mm, 3/8 circle needle, which was manually straightened and introduced percutaneously under laparoscopic control. With the aid of laparoscopic forceps, the needle was punctured outwards, creating the U-suture (Supplementary Materials, Video S7). Two additional suture loops combining the use of EndoClose and NSLT were necessary for partial closure of the IIR. The contralateral vaginal ring was closed with a single suture loop using NSLT as prophylaxis. The duration of anesthesia and surgery were 120 and 95 min.

Three months after hospital discharge, no re-herniation had occurred and the animal maintained normal sexual behavior.

## 4. Discussion

This article describes, for the first time, the LAPS technique of the IIR for repair of inguinal/scrotal hernias in rams. Traditionally, surgical treatment of IH in small ruminants has been performed by complete herniorrhaphy of the external inguinal ring, after castration. If the testicle must be spared, the surgical technique of choice is to partially close the IIR, which requires the use of laparoscopic surgery. The veterinary species in which the largest number of laparoscopic techniques have been described for this purpose is the horse, for which we can find several different approaches [4,16,17].

The cost of laparoscopic procedures is often prohibitive for ruminant livestock. Despite this fact, different laparoscopic techniques have been reported in these animals [11,12,15,18,19,20,21,22]. There are also two different case reports on rams with IH surgically treated by laparoscopic techniques. In the first of them, the authors combine different laparoscopic techniques described in other species, including the placement of polypropylene retroperitoneal meshes in a single case. Most of the techniques employed in this case require advanced laparoscopic training and/or the use of expensive consumables [23]. In the second, most recent, case report, authors used barbed sutures [24] in a ram. This is a technique used in horses [25] which is simple to apply if using the EndoStitch device (Medtronic, Madrid, Spain), but can be technically more demanding if used with needle holders as described in this case report of one ram.

In the present study, we used a technique based on a laparoscopic methodology developed in human pediatrics [5,6] and which has also been successfully used in dogs [8] and pigs [9]. The advantage of this approach is that it reduces the duration of surgery, avoids the use of specific high-cost consumable materials and, fundamentally, it can be performed without the use of complex laparoscopic techniques, as it is based on extracorporeally applied knots [7,8,9]. However, minimal laparoscopic equipment is still needed (laparoscope, laparoscopic cannula and forceps) and, although this type of material is often available in large animal hospitals (mainly for use in horses), this may be limiting in small ruminant practice. Nevertheless, and as mentioned previously, laparoscopic techniques are being developed in this species for different purposes such as artificial insemination [10], which may make this equipment available to some ovine veterinarians.

Despite all the important advantages that laparoscopy can bring to small ruminant surgery, laparoscopic procedures in sheep present some adjunctive complications related to the placement of the first trocar, due to the possibility of accidentally puncturing the rumen or entering the omental bursa [24]. To reduce the incidence of such unwanted events, a fasting period of at least 24 h prior to surgery is usually carried out. This could not be performed in the cadaveric study presented here and, actually, in one cadaver, there was a ruminal perforation and, in another animal, the second trocar entered the omental pouch. Such events did not occur in any of the three clinical cases, all of which included a fasting period. Additionally, regarding fasting to reduce the size of the rumen, the use of the EndoTIP (also known as Ternamian cannula) allows visually controlled insertion of this trocarless helical optical cannula and can minimize the incidence of such complications [26]. In two of the three patients, the surgery was simple and fast to perform, without intraoperative complications except for a slight tearing of the peritoneum adjacent to the vaginal ring that occurred in Case 3. In our own experience with other species, these small peritoneal lacerations adjacent to the vaginal ring usually heal without causing problems or forming adhesions [16]. In Case 2, another complication took place: a subcutaneous and retroperitoneal emphysema formed and prevented visibility; thus, it was not possible to safely performing the LAPS of the IIR. It could be thought that this complication is not directly related to the LAPS technique but to the laparoscopic procedure itself. However, it may also be possible that the formation of retroperitoneal emphysema was related to the incision over the inguinal ring and the first attempts to pass the needle percutaneously.

In the development of a novel surgical technique, it is necessary to carry out pilot trials in order to be able to apply such technique in clinical cases, which is why many authors include in their work preliminary studies in which only experimental animals or cadavers are used [13,27]. In our work, the described technique was performed on cadavers prior to the application to three clinical cases of rams with IH.

As a result of the trial in cadavers, although three laparoscopic portals were placed in all of them, we believe that the procedure could be performed using only one portal for the optics, or with only one extra portal for instruments. In our hands, this was facilitated when using the EndoClose device. In the three clinical cases, however, having two work portals facilitated the hernia’s reduction and LAPS maneuvers in both inguinal rings. In our opinion, the advantage of operating with only an optical portal is the main justification for using the commercial EndoClose device instead of the NSLT. However, as shown in dogs [8] and pigs [9], the LAPS technique can be performed without any instrumental portal, even when using NSLT.

According to our experience, the use of EndoClose does not bring any other additional benefit and increases the cost of the technique. In addition to being a more economic option, an additional advantage of NSLT over EndoClose is that it could facilitate making several entries with the needle around the margins of the ring to pass the suture several times through the same loop, thus creating a purse-string suture as described in children [28]. This could be particularly useful when the ring is very large and requires several knots, to avoid having to pass several loops. However, a limitation of this study is that the cadaveric study was performed on animals without IH and with IIRs of smaller size than those observed in the three clinical cases. In these patients, however, we chose to use the U-suture and not the purse-string stitch described in human medicine. The rationale for this choice was to better control the tightness of the partial closure to prevent compromising testicular functionality. Thus, in Cases 1 and 3, up to three U-sutures were used for an adequate partial closure of the herniated IIR while in the original technique reported in children, only one loop is used, but with the aforementioned purse-string pattern [28]. Works focusing on dogs and pigs using this LAPS technique [8,9] also employed a U-suture, but in all cases, only one loop was used, without the need for additional sutures. This may be due to smaller IIR size in these species compared to sheep, or even to the greater experience of surgeons in previous animal studies.

Another relevant point is the non-absorbable nature of the material used for suturing in this study. Several studies on laparoscopic hernioplasty in humans [5], dogs [8] and horses [4,16] recommend this type of non-absorbable material to maintain a mechanical closure over time, as this could provide additional security in the case of very large IIRs. Laparoscopic follow-up at 4 weeks in such studies showed no deleterious effects derived from the use of non-absorbable sutures such as infections, adhesions or other foreign body reactions [16]. However, good results have also been obtained in porcine species with this technique, using absorbable sutures [9]. Further studies can help to clarify the most suitable material in the longer term. The clinical cases described here presented acquired hernias for which it was not possible to identify a traumatic event that caused them. However, acquired traumatic scrotal hernias are usually much more common in rams than congenital hernias [23]. Although the heritability of this condition is not well-understood, it is imperative to properly inform owners of affected animals if they intend to use them as breeding rams [1]. In those cases where reproductive activity is to be maintained after surgery, it is imperative that the partial closure of the canal does not affect testicular perfusion and functionality. In the case described by Daniel et al. in 2015 [23], the animal was previously castrated from the unaffected testicle and sperm production of the affected testicle could be monitored up to 5 months post-surgery. In our Cases 1 and 3, a specific study of reproductive functionality was not carried out. The impossibility of long-term reproductive monitoring, the non-blinded interpretation of results, and the small number of animals constitute the main limitations of our study.

Another limitation to this study was that a control group was not included to compare the outcome of the new technique. However, it should be noticed that there are currently no standardized or preferred techniques for partial closure of IIRs in rams, with only two single-animal reports so far. Therefore, this study is presented as a first step to describe the technique with the aims of developing it further and comparing it with other methodologies according to the advancement of the field.

In addition to the value of the LAPS methodology for small ruminant surgery, our results open up the possibility to use this technique in foals with congenital IH. It should be considered that some of the techniques and devices developed for adult horses have not yet been tested in foals or have only been described for standing surgery [4], which is unfeasible with foals. It should also be noted that procedures under general anesthesia in neonates and foals have a higher anesthetic risk than in adult horses [29], so any technique that simplifies the procedure and shortens the intervention is desirable. The potential application of the LAPS technique in equine neonates requires studies in such animals, with a large number of cases to study the efficacy and possible influence on long-term testicular activity.

## 5. Conclusions

Laparoscopically assisted percutaneous IIR suturing is a simple and feasible surgical procedure for rams with inguinal hernias when preservation of testicles is preferred. This laparoscopic hernioplasty technique can be considered as an alternative to herniorrhaphy with orchiectomy in rams. However, larger numbers of cases, as well as prospective, longer and more comprehensive follow-up would be required to evaluate long-term safety and effectiveness.

## Figures and Tables

**Table 1:** Characteristics of ram cadavers used in cadaveric study and findings during procedures with each method employed for laparoscopically assisted percutaneous internal ring suturing.

Order	Age (Months)	Weight (kg)	Incidences Related to Portal Placement	EndoClose ^a^		NSLT ^b^		Total U Sutures
				Side	U-Sutures	Side	U-Sutures	
1	54	75	-	Right	3	Left	2	5
2	48	75	1st portal: rumen puncture	Left	1	Right	2	3
3	60	65	Previous ruminal trocar	Right	1	Left	1	2
4	60	90	2nd portal: into omentum	Right	2	Left	1	3
5	82	65	Previous ruminal trocar	Right	1	Left	1	2
6	36	85	-	Left	1	Right	1	2
Mean	56.7	75.8			1.5		1.3	2.8
SD	15.3	10.2			0.8		0.5	1.2
Total					9		8	17

^a^ Grasping the suture with EndoClose.; ^b^ NSLT: Grasping the suture with a loop of polyamide suture passed thought a 12 G 80 mm needle.

## Data Availability

Data are contained within the article or supplementary material.

## Supplementary Materials

The following supporting information can be downloaded at: https://www.mdpi.com/article/10.3390/ani13050836/s1, Video S1: Main steps of the needle suture loop technique (NSLT) for laparoscopically assisted percutaneous suture of the right internal inguinal ring in a cadaver. Video S2: Procedure to verify the adequacy of a right internal ring hernioplasty in a cadaver. A blunt laparoscopic trocar can be inserted through an incision in the scrotum and tunica vaginalis before the suture is tightened. After tightening and knotting the suture on the outside, it is not possible. Video S3: Laparoscopy of case 1. The hernia could be reduced with the combination of external manipulation, laparoscopic atraumatic grasping traction, Trendelenburg position and capnoperitoneum. Video S4: Laparoscopy of Case 1. The contralateral (right) internal ring showed no signs of herniation, but a single percutaneous U-suture was performed. In this occasion, a needle of 20 G and 70 mm was placed under laparoscopic control as a guide to insert the EndoClose device. Video S5: Laparoscopy of Case 2. To reduce the hernia, traction from the inside using laparoscopic forceps was unsuccessful and it was necessary a vigorous external scrotal massage. The hernia contents were found to include abundant loops of small bowel, omentum and fluid. Video S6: Laparoscopy of Case 3. The hernia was easily reduced by traction of the intestine from the inside with laparoscopic forceps. The amount of herniated bowel was less than in previous cases. Video S7: Laparoscopy of Case 3. A first U-suture was performed using a polyamide 1 USP suture with its own traumatic, 30 mm, 3/8 circle needle, stretched manually in order to obtain a reasonably straight needle and introduced percutaneously under laparoscopic control. Two additional U-sutures combining the use of EndoClose and needle suture loop technique were necessary for partial closure of the inguinal ring.
